# Survey of the incidence and effect of major life events on graduate medical education trainees

**DOI:** 10.3402/meo.v20.27597

**Published:** 2015-06-11

**Authors:** Lars J. Grimm, Alisa Nagler, Charles M. Maxfield

**Affiliations:** 1 Department of Radiology, Duke University Hospital, Durham, NC, USA; 2 Graduate Medical Education, Duke University, Durham, NC, USA

**Keywords:** life events, graduate medical education, support systems, demographics, training, career decision making, institutional infrastructure

## Abstract

**Purpose:**

This study aims to assess the incidence of major life events during graduate medical education (GME) training and to establish any associations with modifiable activities and career planning.

**Methods:**

The authors surveyed graduating GME trainees from their parent institution in June 2013. Demographic information (clinical department, gender, training duration) and major life events (marriage, children, death/illness, home purchase, legal troubles, property loss) were surveyed. Respondents were queried about the relationship between life events and career planning. A multivariable logistic regression model tested for associations.

**Results:**

A total of 53.2% (166/312) of graduates responded to the survey. 50% (83/166) of respondents were female. Major life events occurred in 96.4% (160/166) of respondents. Male trainees were more likely (56.1% [46/82] vs. 30.1% [25/83]) to have a child during training (*p*=0.01). A total of 41.6% (69/166) of responders consciously engaged or avoided activities during GME training, while 31.9% (53/166) of responders reported that life events influenced their career plans. Trainees in lifestyle residencies (*p*=0.02), those who experienced the death or illness of a close associate (*p*=0.01), and those with legal troubles (*p*=0.04) were significantly more likely to consciously control life events.

**Conclusion:**

Major life events are very common and changed career plans in nearly a third of GME trainees. Furthermore, many trainees consciously avoided activities due to their responsibilities during training. GME training programs should closely assess the institutional support systems available to trainees during this difficult time.

*Life is what happens to you while you're busy making other plans.* (1)

Former Beatle John Lennon's famous lyric reflects an undeniable paradox which certainly applies to graduate medical education (GME). The long and intense years of internship, residency, and fellowship can seem all-consuming, but they do not provide immunity from the significant life events which can affect any young adult.

The typical medical student transitions to GME training in their mid- to late-20s, a time when many individuals in society are considering marriage, children, and the purchase of a first home, and some must endure the illness or death of an aging parent (2–4). While some of these life events are unforeseen and uncontrollable, others require conscious planning and execution. There was a time when a resident was expected to live at the hospital, and put the rest of his or her life on hold for the sake of professional immersion (5). With changes in GME expectations over the last century, punctuated by duty hour reforms in 2003 (6) and generational differences, trainees now may be less willing to postpone controllable life events (7–13).

Historically, most reform in GME has focused on the needs of the health care system (14). Increasingly in recent years, focus has turned toward the importance of trainee quality of life (15–18), with an emphasis toward limiting the time residents spend in the hospital (19). In contrast, minimal effort has been directed toward documenting and understanding what important life events are likely to occur during this time and the complex interplay they have with GME training. These life events occur in parallel to the experience and demands of GME training, and they may or may not impact resident education and job performance. It is not necessary for life events to have a significant detrimental effect on resident job performance to be of concern to the training program. Understanding the breadth and depth of life events is important when building support systems for trainees who undergo these challenging experiences. For prospective GME trainees, it is important to understand the likelihood of these life events when evaluating the support systems available at training programs.

To our knowledge, there are no studies in the published literature that have documented the diverse spectrum of life events that occur during GME training. Our study seeks to provide clarity to this issue. The specific goals of our study are to document the incidence of major life events that occur during GME training in a sample of trainees from one academic health science center and to understand how training influences these events and how these events influence training. Findings should inform future and current trainees as well as GME sponsoring institutions on the infrastructure and support needed to maximize the trainee experience, foster quality educational experiences, and guide career planning.

## Methods

### Participants

All residents and fellows (*n*=312) at our institution scheduled to graduate between June 30th and July 31st, 2013 were eligible for inclusion. Individuals comprised a spectrum of 15 different medical departments, including subspecialty fellowships. Our institution is a major academic medical center in a suburban setting in the Southeastern United States. Individuals were identified via e-mailing lists from the GME office.

### Research design

We developed a seven-question online survey for all graduating residents and fellows at our institution scheduled to graduate between June 30th and July 31st, 2013 (Appendix). The survey questionnaire was designed based on input from residents, GME program directors, and the GME office in conjunction with a review of the available published literature. The study was designed to capture a diverse spectrum of life events and their potential influence on GME and future career choices. The study was designed to be conducted as a prospective questionnaire study.

### Survey questionnaire

The survey was distributed in early June 2013. The email included the following message. ‘Please take 5 minutes to complete this brief online survey. We recognize that GME training requirements coupled with life outside of GME can be overwhelming. We are interested in learning more about the major life events that residents and fellows experience during their GME training. This may assist GME programs and institutions in better providing support to residents/fellows to ensure a meaningful and successful experience’. We told recipients that their participation was voluntary and that their responses were not intended to be linked to them in any way. We sent a reminder email 2 and 7 days after the initial email.

Demographic questions included clinical department, gender, and years of GME training. The graduates were asked about their relationship status and whether they had any children upon entering GME training (Table 1). Graduates were then asked to select from a list of major life events that occurred during their GME training (Table 2). We compiled a list of major life events based on informal discussions between the authors and current GME trainees, with the goal of including a broad range of life events which would have a significant effect on the life of an individual. A follow-up question assessed whether there was a conscious decision to engage, or not engage (when not expected or planned), in any of these activities because they were in a GME training program. Finally, they were asked if any of these life events had a direct impact on their career choice or decision to pursue additional GME training. Responders were also given the option to include free text comments for each question which were incorporated into the findings when applicable.

**Table 1:** Distribution of demographic information upon entering GME training

Answer options	Total, no. (%)	Men, no. (%)	Women, no. (%)	*P*
Total	166	82	83	
Single	45 (27.1)	23 (28.0)	22 (26.5)	0.88
Significant other	35 (21.1)	18 (22.0)	16 (19.3)	0.71
Engaged	12 (7.2)	8 (9.8)	4 (4.8)	0.25
Married	74 (44.6)	33 (40.2)	41 (49.4)	0.35
No children	144 (86.7)	68 (82.9)	75 (90.4)	0.16
Had one child	15 (9.0)	8 (9.8)	7 (8.4)	0.80
Had 2 or more children	7 (4.2)	6 (7.3)	1 (1.2)	0.06

**Table 2:** Distribution of life events during GME training

Answer options	Total, no. (%)	Men, no. (%)	Women, no. (%)	*P*
Total	166	82	83	
Change of GME residency (program or institution)	28 (16.9)	15 (18.3)	13 (15.7)	0.68
Relationship status				
Engagement	41 (24.7)	25 (30.5)	15 (18.1)	0.11
Marriage	46 (27.7)	28 (34.1)	17 (20.5)	0.10
Break up with significant other	23 (13.9)	12 (14.6)	11 (13.3)	0.81
Divorce	5 (3.0)	2 (2.4)	3 (3.6)	0.66
Children				
Pregnancy	64 (38.6)	35 (42.7)	29 (34.9)	0.42
Miscarriage	13 (7.8)	4 (4.9)	9 (10.8)	0.17
Birth of one child	45 (27.1)	27 (32.9)	18 (21.7)	0.17
Birth of 2 or more children	26 (15.7)	19 (23.2)	7 (8.4)	0.02*
Adoption of a child	0 (0)	0 (0)	0 (0)	a
Death of a child	0 (0)	0 (0)	0 (0)	a
Death or illness				
Death of a significant other	2 (1.2)	1 (1.2)	1 (1.2)	0.99
Death of a close friend	21 (12.7)	9 (11.0)	12 (14.5)	0.53
Death of a close family member	55 (33.1)	29 (35.4)	26 (31.3)	0.65
Personal illness or injury requiring leave from work	13 (7.8)	5 (6.1)	8 (9.6)	0.42
Significant other/spouse – death of a close family member	23 (13.9)	11 (13.4)	12 (14.5)	0.86
Leave of absence for illness in family member	9 (5.4)	5 (6.1)	4 (4.8)	0.72
Financial				
Significant other/spouse – loses job	13 (7.8)	3 (3.7)	10 (12.0)	0.05*
Significant other/spouse – start new job and/or change of career plan	52 (31.3)	25 (30.5)	27 (32.5)	0.81
Violation of the law/arrest/imprisonment of family member or close friend	6 (3.6)	4 (4.9)	2 (2.4)	0.40
Purchase of a home	70 (42.1)	31 (37.8)	39 (47.0)	0.36
Lawsuit	3 (1.8)	1 (1.2)	2 (2.4)	0.57
Major property damage/loss	12 (7.2)	3 (3.7)	9 (10.8)	0.08

a No *p-*value due to zero incidence.

* *P*≤0.05.

### Data analysis and management

We calculated the distribution of answers in total and for both genders, with differences compared via a Chi-squared analysis. To assess whether there was any influence on the conscious decision during GME training to engage in certain activities, as well as on career choices or additional training a multivariate logistic regression model was created. The study was designed to detect a moderate effect size (f^2^=0.2) with a power of 0.8 and *p*-value of 0.05 considered statistically significant. As part of this analysis, several variables were combined due to the low incidence/prevalence (Table 3). GME training programs were categorized into surgical (orthopedic surgery, general surgery, ophthalmology), lifestyle (anesthesiology, dermatology, pathology, radiology, radiation oncology), and clinical (community and family medicine, internal medicine, neurology, obstetrics and gynecology, pediatrics, psychology, combined programs). We performed the statistical analysis utilizing JMP Pro (version 9.0.0, SAS Institute Inc., Cary, NC). We considered a *p*-value <0.05 as statistically significant.

**Table 3:** Multivariate analysis comparing life events to a conscious decision to engage in activities as well as career planning

	Conscious decision to engage in life events		Influence on career planning or training	
	
Life event	Odds ratio (95% CI)	*P*	Odds ratio (95% CI)	*P*
Residency type				
Lifestyle	1.00 (reference)		1.00 (reference)	
Surgical	0.27 (0.08–0.83)	0.02*	0.97 (0.30–3.01)	0.96
Clinical	0.58 (0.25–1.28)	0.18	1.26 (0.56–2.99)	0.58
Gender				
Male	1.00 (reference)		1.00 (reference)	
Female	1.15 (0.56–2.35)	0.70	1.57 (0.77–3.24)	0.21
Relationship upon entering GME training				
Single	1.00 (reference)		1.00 (reference)	
Not single	1.49 (0.66–3.44)	0.34	0.63 (0.28–1.41)	0.26
Child status upon entering GME training				
None	1.00 (reference)		1.00 (reference)	
Children	0.45 (0.14–0.31)	0.15	1.00 (0.31–2.94)	1.00
Change in GME training program				
No	1.00 (reference)		1.00 (reference)	
Yes	1.18 (0.46–2.99)	0.73	1.12 (0.44–2.73)	0.80
Got engaged or married during GME training				
No	1.00 (reference)		1.00 (reference)	
Yes	0.60 (0.27–1.28)	0.19	1.42 (0.67–3.02)	0.36
Got divorced or broke up during GME training				
No	1.00 (reference)		1.00 (reference)	
Yes	0.73 (0.27–1.88)	0.52	0.68 (0.24–1.73)	0.43
Major death or illness				
No	1.00 (reference)		1.00 (reference)	
Yes	4.17 (1.52–12.6)	0.01*	0.87 (0.30–2.28)	0.78
Major financial change				
No	1.00 (reference)		1.00 (reference)	
Yes	1.53 (0.74–3.19)	0.25	1.26 (0.61–2.67)	0.54
Legal troubles				
No	1.00 (reference)		1.00 (reference)	
Yes	6.69 (1.10–61.3)	0.04*	0.90 (0.12–4.52)	0.90

* *P*<0.05.

### Human subjects review

The study was granted an institutional review board exemption and was in compliance with the Helsinki Declaration.

## Results

**Fig. 1:**
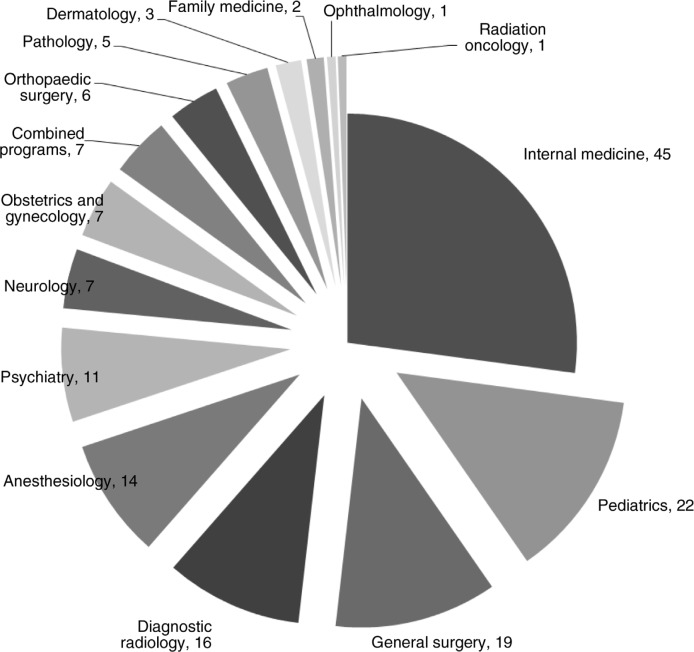
Distribution of responders by graduate medical education programs.

One hundred and sixty-six (166/312, 53.2%) individuals from 15 specialties completed the survey. The distribution of GME programs represented is shown in Fig. 1. Fifty percent (83/166) of responders were women and 49.4% (82/166) were men, with one non-responder. The average number of years of GME training was 5.2 years±1.7 (standard deviation; range: 3–12). Upon entering GME training, responders were most commonly married (44.6%, 74/166) and without children (86.7%, 144/166). There was no significant difference in the distribution of these variables between male and female respondents as shown in Table 1.

Major life events occurred in 96.4% (160/166) of trainees. The only significant differences between gender were that men were more likely than women to have two or more children (23.2% vs. 8.4%, *p*=0.02) and women were more likely to report a spouse losing a job (12.0% vs. 3.7%, *p*=0.05). The distribution of specific events is shown in Table 2.

Of all responders, 41.6% (69/166) reported that there was a conscious decision to engage or not engage in major life events during GME training with several variables demonstrating significance on the multivariable logistic regression model (Table 3). Many responders provided free text (*n*=62) comments describing how their GME training influenced their ability to engage in life events. A representative sample of comments is shown in Table 4. GME trainees in surgical programs were less likely to consciously engage in major life events compared to trainees in lifestyle programs (odds ratio [OR] 0.27, 95% confidence interval [CI] 0.08–0.83, *p*=0.02). For example, a male ophthalmology respondent wrote that, ‘I tried to prevent my GME responsibilities (from) interfering with my personal life whenever possible’. Trainees who experienced the death or major illness of a close friend or family member were significantly more likely to consciously engage in major life events (OR 4.17, 95% CI 1.52–12.6, *p*=0.01). Finally, those trainees that experienced legal troubles were significantly more likely to consciously engage in major life events (OR 6.69, 95% CI 1.10–61.3, *p*=0.04). A common theme among the free text comments related to having children and marriage which was prevalent for both male and female respondents. A female community and family medicine respondent wrote that, ‘I did not pursue having further children in residency because of how unsupportive I saw my program as being’, while a male internal medicine respondent wrote that ‘I had to make a somewhat faster decision to get married than we probably would have done otherwise, simply because I had only one long consolidated vacation period in which we could have gotten married’.

**Table 4:** A selection of open-ended comments provided by respondents

**Was there a conscious decision to engage (or not) in life events during GME training?**
In regards to my parent's illness, I was essentially not able to be present for it to provide support. (male)
When I arrived at fellowship, we began trying to get pregnant and ultimately realized we were dealing with infertility. I don't know how my decision to push off starting a family for 4 years really effected this (whether I would have had problems anyway), but it was the hardest thing I have ever been through. (female)
Getting time off from residency and fellowship for major life events can be politically damaging in my opinion …. Program leadership or chief residents can look at asking for time away as a ‘weakness’. (male)
I chose not to go to my grandfather's funeral. (male)
I decided not to become pregnant during residency. I felt that pregnancy was not encouraged/supported in my program. (female)
I delayed marriage until I was at the cusp of completing my training and already had a job lined up. (male)
**Did life events have impact on career choice or whether to pursue further GME training?**
Having kids made me lean away from academics to pursue a job with more vacation and money and less after-hours investment. (male)
I will likely not go into fellowship because I want to spend more time with my young children. (male)
The birth of my child will likely prevent advanced fellowship training due to poor compensation of trainees. (male)
Greeting married, having a stepchild and having two loved ones die in residency re-focused my priorities (female)
Chronic illness diagnosis affected plans for fellowship (male)
My life has evolved around my chosen career … kinda sad (female)

A total of 31.9% (53/166) of all respondents reported that a major life event had a direct impact on his/her career choice or the decision to pursue additional GME training. This distribution was not significantly different between men and women (26.8% vs. 36.1%, *p*=0.29). None of the variables assessed demonstrated a significant influence on the multivariable logistic regression model (Table 3). Many of the free text comments from both men and women directly addressed the issue of how training has influenced future career choices specifically regarding the influence of children and low pay in academics or during fellowship training. A female obstetrics and gynecology respondent wrote that, ‘I am not pursuing additional GME training so that I can finally focus on my marriage and my child in a manner that I am happy with’. While a male medicine respondent wrote, ‘More training would limit (my) ability to financially support (my) significant other in the short term, making prolonged training less desirable’.

## Discussion

Our study provides insights into the incidence of major life events occurring during GME training, and their effect on trainees, albeit at a single tertiary care academic center. The vast majority of trainees (96.4%) reported at least one major life event during their GME training: 27.7% of trainees were married during their GME training; 42.8% experienced the birth of a child during training; and 47.0% endured the death of a significant other, close friend, or family member during GME training. Even major life events that are deemed positive, such as the birth of a child or purchase of a home, may be stressful and associated with significant new responsibilities that require a readjustment in the work-life balance for the trainee. Nearly one third (31.9%) of trainees reported that decisions regarding future career choices and additional GME training were impacted by these major life events.

In our study, roughly half of responders entering GME training were married or engaged with a slightly higher rate for women (54.2%) than for men (50.0%). However, during GME training there was a great disparity in the number of new engagements and marriages between men (64.6%) and women (38.6%). Prior studies in the surgical literature have shown a higher incidence of marriage for male versus female trainees, which is borne out in our study which crosses medical disciplines (20, 21). Previous authors have reported that female trainees worry about how they might be perceived by senior residents if married (20). Fortunately, the rate of divorce among our respondents was low at 3.0%, compared to the national average of 5.8% for individuals aged 25–34 years (2). It is hard to put into context the rate of break up with a significant other of 13.9% that we found in the study, as no real comparator data are available; however, there is evidence in the social sciences literature to suggest that lack of a social support network is a robust predictor of non-marital romantic relationship dissolution (22). This suggests that trainees who migrate to a training location without support systems in place are at higher risk of break up. Several of the male respondents in the free text comments reported that they either delayed marriage or had very narrow windows in order to get married due to scheduling of vacations. GME programs may wish to consider added flexibility in scheduling vacation or the ability to bank vacation time in order to accommodate trainees who wish to get married, recognizing that they may need two consecutive weeks of vacation time.

Similar to marriage, having children can also be a source of great joy and stress. Upon entering training, 17.1% of men and 9.6% of women reported having children, but during training 56.1% of men and 30.1% of women reported the birth of one or more children. Most striking was that 23.2% of men reported the birth of two or more children, compared to only 8.4% of women. This gender dichotomy is similar to that reported by prior authors (20, 21), and may be most reflective of the role of the trainee's partner and indicative of more traditional gender roles in which male trainees may be reliant on partners who do not work to assume childcare duties (23–25). Several female trainees reported in the free text comments that they delayed having children during residency because they felt they would not have adequate support, but no such comments were reported by male trainees. Work by prior authors has shown that female residents with children do not look forward to work and are more likely to feel overwhelmed (20, 21). However, recent work has demonstrated that attrition rates were not influenced by child rearing regardless of gender (13). Training programs should consider implementing additional support systems for trainees or spouses who are pregnant, or doing a better job of advertising existing infrastructure, so that trainees feel they are adequately supported during such a stressful time period.

Trainees also experienced a sizeable number of unplanned negative life events, including deaths, legal troubles, illness or injury, property loss, and lose of a job. Almost half of respondents reported the death of a friend or family member. In the multivariable logistic regression model, both death/illness and legal troubles were significantly associated with consciously modifying behavior in relation to major life events. This suggests that the influence of major negative life events has a lasting impact on the way in which the trainees plan the remainder of their time in GME training. In the free text comments, many trainees reported that they did not feel comfortable taking time off to attend funerals or visit family members who were seriously ill because they felt that training programs would not be supportive. Training programs should ensure that they have adequate coverage systems in place to accommodate unexpected life events so trainees are not in the position of having to decide between attending a funeral or providing clinical coverage. The high percentage of trainees with major life events indicates that pulling one trainee to cover for another will likely be reciprocated at a future date.

It is somewhat surprising that GME trainees in surgical fields are significantly less likely to consciously engage, or not engage, in activities while they are in training. The increased time demands placed on surgical trainees while at work may not allow for sufficient time to plan for events outside of work. They may not even have the luxury to think about such events as getting married or buying a house. In contrast, trainees in lifestyle fields may be attracted to those fields because of the added control they will have over their lives when in practice, which in turn may carry over to their time spent during GME training.

The influence of these major life events on future career choices or additional training was quite significant with nearly one third of respondents indicating that it had influenced them. Although none of the variables from the multivariable logistic regression model were predictive, in the free text comments the most common theme was the decision not to pursue an academic career or additional subspecialty training in order to spend more time with family. This opinion was shared fairly evenly between male and female respondents. The belief that advanced training or an academic career negatively impacts family life may be the result of financial constraints that handicap many trainees (26). The large burden of educational debt and the relatively low income during GME training results in trainees feeling pressured to focus on monetary goals. It is hard to dismiss these very real world concerns, but the end result is the potential loss of talented individuals who may have made meaningful contributions to the fields of academic medicine. A medical education system which takes into account these realistic challenges and life events may mitigate stress and foster advanced training and academic careers for individuals. In addition, programs may wish to strongly consider moonlighting opportunities for senior residents and fellows. Individuals may be more inclined to pursue additional training if the pay difference between advanced training and practice is reduced, even if only by a small amount.

With all of the attention focused recently on resident wellness and duty hour restrictions, our research suggests that significant efforts should be undertaken to develop systems to support trainees when they experience major life events. Unplanned life events require formalized support systems provided by the training programs or GME sponsoring institutions. Training programs must have in place the capacity to provide coverage for trainees when they need to be excused unexpectedly. Furthermore, although medical students have been shown to focus more on work culture, collegiality, and program location than reputation rank or job prospects (27), an underappreciated variable may be the degree of support available to the future trainee. Proximity to family and friends are important complements to the formal support system infrastructure provided by training programs.

## Limitations

The limitations of our study primarily stem from the single institution and retrospective study design. Trainees from other institutions may experience different rates of major life events which limit the generalizability of our study. Surveying GME graduates from different parts of the country may reveal interesting geographic trends. The study was designed based on the input of many individuals with different degrees of influence on GME, but the study was not formally pretested. Additionally, the retrospective nature of our study induces a response bias. Graduates with very strong opinions, good or bad, may have been more likely to respond than those without strong opinions which may have influenced our response rates. Prospectively following trainees over time and recording events periodically could provide a more real world snapshot of trainee life events. The major life events chosen for inclusion in this study were based on informal discussions between the study authors and current GME trainees. The inclusion or exclusion of different life events would have an effect on the percentage of trainees experiencing life events. The distribution of graduates by training program is broad but not particularly deep, and many departments, by virtue of the low number of graduates, did not have large sample sizes in our study. Incorporation of multiple institutions or data from multiple years may compensate for this deficit.

## Conclusion

Major life events impact nearly all GME trainees. Such events, good and bad, planned and unplanned, can impact one's educational experience, job performance, and future career decisions. Our data, drawn from a single institution, invites other institutions to perform their own surveys, and to consider the development of infrastructure to support their trainees during challenging times. Furthermore, medical students and residents entering fellowships may wish to consider the strength of their support systems when choosing their future GME training programs.

## Appendix

**1. Within which Department is your GME Program?**

 Anesthesiology         Community and Family Medicine

 Dermatology          Medicine

 Neurology           Obstetrics & Gynecology

 Ophthalmology         Orthopaedic Surgery

 Pathology           Pediatrics

 Psychiatry          Radiation Oncology

 Radiology           Surgery

 Combined (Med-Psych and Med-Peds)

**2. What is your gender?**

 Man         Woman

**3. How many total years of Graduate Medical Education (GME) training have you completed? For this question and all questions in this survey, please consider your total time during all residencies and fellowships (any GME training following medical school)**

3   4   5   6   7   8   9   10   11   12    > 12

**4. When you first entered GME training, which of the following applied to you? (check all that apply)**

 Single

 Single

 Significant other

 Engaged

 Married

 Other (please specify)

 Had one biological child

 Had 2 biological children

 Had 3 or more biological children

 Had one adopted child

 Had 2 adopted children

 Had 3 or more adopted children

**5. Which of these major life events did you experience during your GME training? (check all that apply)**

 Change of GME residency (program or institution)

 Engagement

 Marriage

 Break-up with significant other

 Divorce

 Pregnancy (for you or significant other/spouse)

 Miscarriage (for you or significant other/spouse)

 Birth of one child

 Birth of 2 or more children

 Adoption of one child

 Adoption of 2 or more children

 Death of a child

 Death of a significant other

 Death of a close friend

 Death of a close family member

 Personal illness or injury requiring leave of absence from work

 Significant other/spouse – death of a close family member

 Leave of absence for illness in family member

 Significant other/spouse – loses job

 Significant other/spouse – start new job and/or change of career plan

 Violation of the law/arrest/imprisonmentof family member or close friend

 Purchase of a home

 Lawsuit (medical or non-medical)

 Property loss (due to house fire, car accident, storm damage)

 Other (please specify)

**6. For those which you had control, was there a conscious decision to engage (or not) in the above activities because you were in a GME Program?**

 Yes

 Yes

 No

 Please elaborate:

**7. Did the above life events have a direct impact on your career choice or decision to pursue additional GME training?**

 Yes

 Yes

 No

 Please elaborate:
